# Poststroke neutrophil count is predictive of the outcomes of large-artery atherosclerotic stroke and associated with craniocervical atherosclerosis

**DOI:** 10.1038/s41598-023-37815-5

**Published:** 2023-07-17

**Authors:** Yi Yang, Yue He, Yuhao Xu, Wei Han, Tian Zhao, Yuanwei Shao, Ming Yu

**Affiliations:** 1 Department of Neurology, Affiliated Hospital of Jiangsu University, Jiangsu University, No.438 Jiefang Street, Zhenjiang, Jiangsu China; 2 Department of Radiology, Affiliated Hospital of Jiangsu University, Zhenjiang, China

**Keywords:** Biomarkers, Diseases, Neurology, Risk factors

## Abstract

Elevation of the neutrophil count is detrimental to the outcome of patients with stroke. The effect of poststroke neutrophil count on the outcome of patients with large-artery atherosclerosis (LAA) stroke is unclear. This study aims to explore the relationship of poststroke neutrophil count with the functional outcome of patients with LAA stroke, and the relationship of poststroke neutrophil count and craniocervical atherosclerotic stenosis (AS) number in these patients. The AS was defined as ≥ 50% stenosis or occlusion attributed to atherosclerosis on craniocervical large arteries. A total of 297 participants were enrolled in the cohort. In multivariable analyses, neutrophil count [adjusted relative risk (aRR) = 1.23, 95% confidence interval (CI) 1.09–1.40, *p* = 0.001] was an independent predictor of 90-day poor functional outcome [modified Rankin Scale (mRS) > 2 points]. The neutrophil count was significantly associated with the craniocervical AS number in a multivariable ordinal logistic regression analysis [adjusted odds ratio (aOR) = 1.41, 95% CI 1.16–1.72, *p* = 0.001]. The poststroke neutrophil count is a valuable predictor of 90-day poor functional outcome of patients with LAA stroke. The poststroke neutrophil count is positively correlated with the craniocervical AS number in these patients.

## Introduction

Craniocervical atherosclerotic stenosis (AS), namely ≥ 50% stenosis or occlusion caused by atherosclerosis on craniocervical large arteries, is one of the major causes of acute ischemic stroke. A large body of evidence has demonstrated that the presence of and an increasing craniocervical AS number are detrimental to the functional outcomes and long-term prognoses of patients with stroke^1–7^. In a previous work, we used a semiquantitative method to quantify craniocervical atherosclerotic severity in patients with large-artery atherosclerotic (LAA) stroke, and found that the risk of poor 90-day functional outcome significantly increased along with the worsened craniocervical atherosclerotic severity in these patients^8^. However, the underlying mechanism is still unclear.

Neutrophils are immune cells with an early response to the onset of stroke. Within a few hours post-stroke onset, neutrophils significantly increase in the peripheral circulation^9^, migrate into the cerebral pia mater and perivascular spaces and ultimately infiltrate into the ischemic parenchyma^10^. Upon arrival, neutrophils release substantial toxic cytokines and mediate subsequent migration of inflammatory or immune cells to the ischemic lesion^11^. Furthermore, neutrophils can breakdown the blood‒brain barrier and promote thrombosis through various pathways, including the formation of neutrophil extracellular traps and the release of a series of cytokines, such as cathepsin G, elastase, and matrix metalloproteinases^11–14^. Consequently, an elevation of poststroke neutrophil count is adverse to the functional outcome of patients, which has been corroborated by a series of clinical studies^15–17^. However, these works did not investigate the association between the poststroke neutrophil count and the clinical outcomes of patients with LAA stroke.

A previous community-based study reported that the neutrophil-to-lymphocyte ratio is positively associated with intracranial atherosclerotic severity^18^. However, that study enrolled only healthy participants and did not investigate the relationship of the neutrophil count with atherosclerotic severity post stroke onset. Furthermore, that study did not analyze the correlation of neutrophil count with the comprehensive condition of craniocervical atherosclerosis. Quan and colleagues found that patients with LAA stroke had a higher poststroke neutrophil-to-lymphocyte ratio than those with the small vessel occlusion subtype^19^, suggesting that poststroke neutrophil count is likely associated with craniocervical atherosclerotic severity. Nevertheless, this study did not further analyze the correlation of neutrophil count with craniocervical AS number in the LAA stroke population. We speculate that the poststroke neutrophil count is likely to increase with an increasing craniocervical AS number in patients with LAA stroke. Given the injuries that neutrophils cause to the ischemic parenchyma, our conjecture could contribute to interpreting the adverse effect of craniocervical atherosclerotic burden on the outcomes of patients with LAA stroke.

The present study aims to investigate the predictive value of poststroke neutrophil count for the 90-day poor functional outcome in patients with LAA stroke and to analyze the relationship between the poststroke neutrophil count and the craniocervical AS number to explore a valuable predictor of poor outcome in patients with LAA stroke and to interpret the underlying mechanism of the association between the craniocervical atherosclerotic burden and the poor outcome of patients with LAA stroke.

## Methods

### Study populations

This prospective single‒center study adhered to STROBE guidelines. This study was in accordance with The Code of Ethics of the World Medical Association (Declaration of Helsinki) and was approved by the Medical Research Ethics Committee of Affiliated Hospital of Jiangsu University. Informed consent was obtained from all study subjects. Patients admitted to the stroke unit within 7 days of onset between August 01, 2021, and July 31, 2022, and diagnosed with acute ischemic stroke were consecutively enrolled in this study. The inclusion criteria were age ≥ 18 years old and diagnosed with LAA stroke according to the Trial of Org 10,172 in Acute Stroke Treatment^20^. Patients lacking neuroimaging or routine blood test data, with poor neuroimaging quality or diffusion-weighted image (DWI) negative stroke, malignant tumor, connective tissue diseases, hematopathy, infection, ischemic or hemorrhagic stroke within 6 months before the index stroke, organ failure, immunosuppressor use or a prestroke mRS > 2 points were excluded.

### Clinical baseline characteristics

All patients underwent routine blood and high-sensitivity C-reactive protein (hs-CRP) tests within 2 h post admission to the emergency center or the stroke unit. After admission, the clinical baseline characteristics were collected by a face-to-face interview and included age, sex, hypertension and diabetes mellitus history, smoking, alcohol consumption, previous stroke (ischemic or hemorrhagic stroke) and medication use. Smoking was considered ≥ 1 cigarette per day for at least 6 months^21^. Alcohol consumption was considered as drinking > 2 U/day on average for men or > 1 U/day on average for women^22^. The neurological deficit severity of each patient was evaluated by an experienced neurologist according to the National Institute of Health Stroke Scale (NIHSS)^23^. All patients were treated based on the updated acute ischemic stroke management guidelines^21^.

### Evaluation of craniocervical AS

Computed tomography angiography (CTA) was performed to evaluate craniocervical atherosclerosis. The intracranial large arteries under evaluation included the bilateral anterior cerebral (A_1_/A_2_ segments)_,_ middle cerebral (M_1_/M_2_ segments), posterior cerebral (P_1_/P_2_ segments), intracranial internal carotid, intracranial vertebral arteries, and basilar artery. The evaluation of intracranial stenotic degree was consistent with the Warfarin-Aspirin Symptomatic Intracranial Disease Study Trial method^24^. The extracranial large arteries under evaluation included the bilateral common carotid, extracranial internal carotid, and extracranial vertebral arteries. The evaluation of extracranial stenotic degree was consistent with the North American Symptomatic Carotid Endarterectomy Trial method^25^. The definition of AS was ≥ 50% stenosis or occlusion attributed to atherosclerosis on craniocervical large arteries^7^. The total number of craniocervical large arteries with an AS in each participant was counted as the craniocervical AS number. In the case of tandem stenoses situated on a single large artery, the craniocervical AS number was counted as 1.

Craniocervical AS was evaluated by two independent and experienced radiologists blinded to the participants’ clinical baseline characteristics. If there were disagreements in their evaluations, a third superior expert made the final decision. Cohen’s kappa was used to determine the intraobserver and interobserver agreements in the evaluation of craniocervical AS^26^. The κ values of the intraobserver and interobserver agreements were 1.00 and 0.794, respectively (both *p* < 0.001).

### Measurement of the infarct size

**Figure 1:**
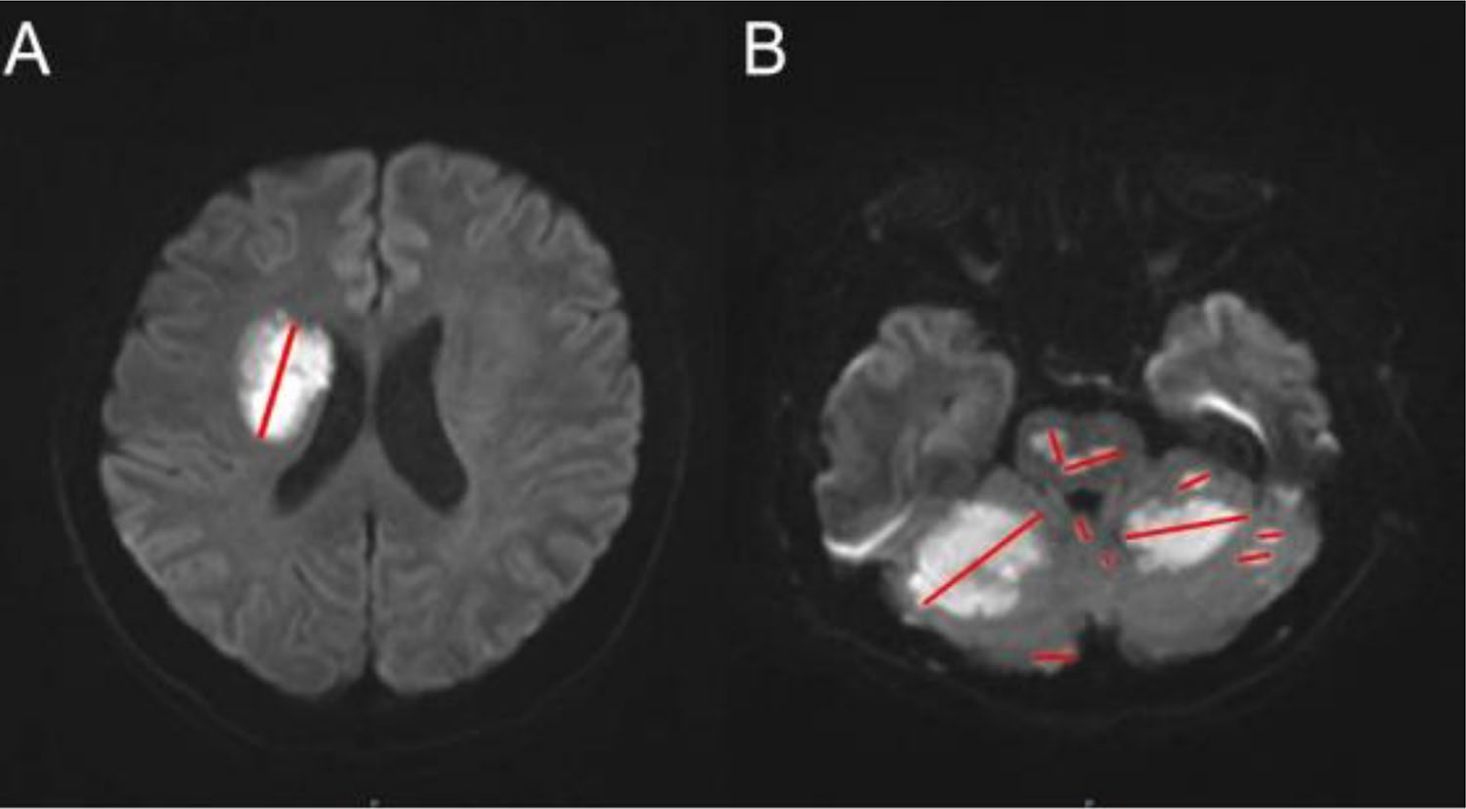
Graphical representation of the measurement of the infarct size. (**A**) single lesion; (**B**) multiple lesions: infarct size was represented with the sum of all largest lesional diameters.

A previous study reported that poststroke neutrophil count was significantly associated with infarct size^27^. Therefore, the present study measured participants’ infarct sizes, which might act as a confounder influencing the correlation of poststroke neutrophil count with craniocervical AS number. Infarct size was represented by the largest lesional diameter on the section with the largest infarct in DWI, as previously reported^28^. In the case of multiple infarcts, infarct size was defined as the sum of the largest diameters of all lesions (Fig. 1).

### Follow-up

An independent neurologist blinded to the participants’ clinical information contacted the participants or surrogates via telephone at 30, 60 and 90 days post onset of the index stroke to understand the control of risk factors and medication compliance. At the 90-day follow-up, participants’ daily activities were evaluated according to the modified Rankin Scale (mRS) (0 ~ 5 points, death was counted as 6 points)^29^. A 90-day poor functional outcome was considered an mRS > 2 points at the 90-day follow-up.

### Statistical analysis

The chi‒square test or Fisher’s exact test was used in the comparisons of categorical variables. The Shapiro‒Wilk test was used to test the data distribution. Normally distributed continuous variables are described by the mean ± standard deviation (SD), and nonnormally distributed continuous variables are described by the median (interquartile range, IQR). Student’s *t* test or Mann‒Whitney *U* test was used in the comparisons of continuous variables between 2 groups, whereas the one-way analysis of variance test or Kruskal‒Wallis test was used in the comparisons of continuous variables among 3 groups, if appropriate.

After the univariable analysis, age, sex and clinical factors with a *p* value < 0.1 were included in the multivariable modified Poisson regression analysis^30^ to determine the relationships of craniocervical AS number and poststroke inflammatory markers with 90-day poor functional outcome. Subsequently, all participants were categorized into 3 groups based on the tertiles of the craniocervical AS number. Multivariable ordinal logistic regression analysis was used to determine the relationship of the poststroke inflammatory markers with the craniocervical AS number. All statistical analyses were performed by using SPSS software 25.0 (IBM, Armonk, NY, United States). All tests were two-sided, and a *p* value < 0.05 was considered statistically significant.

## Results

**Figure 2:**
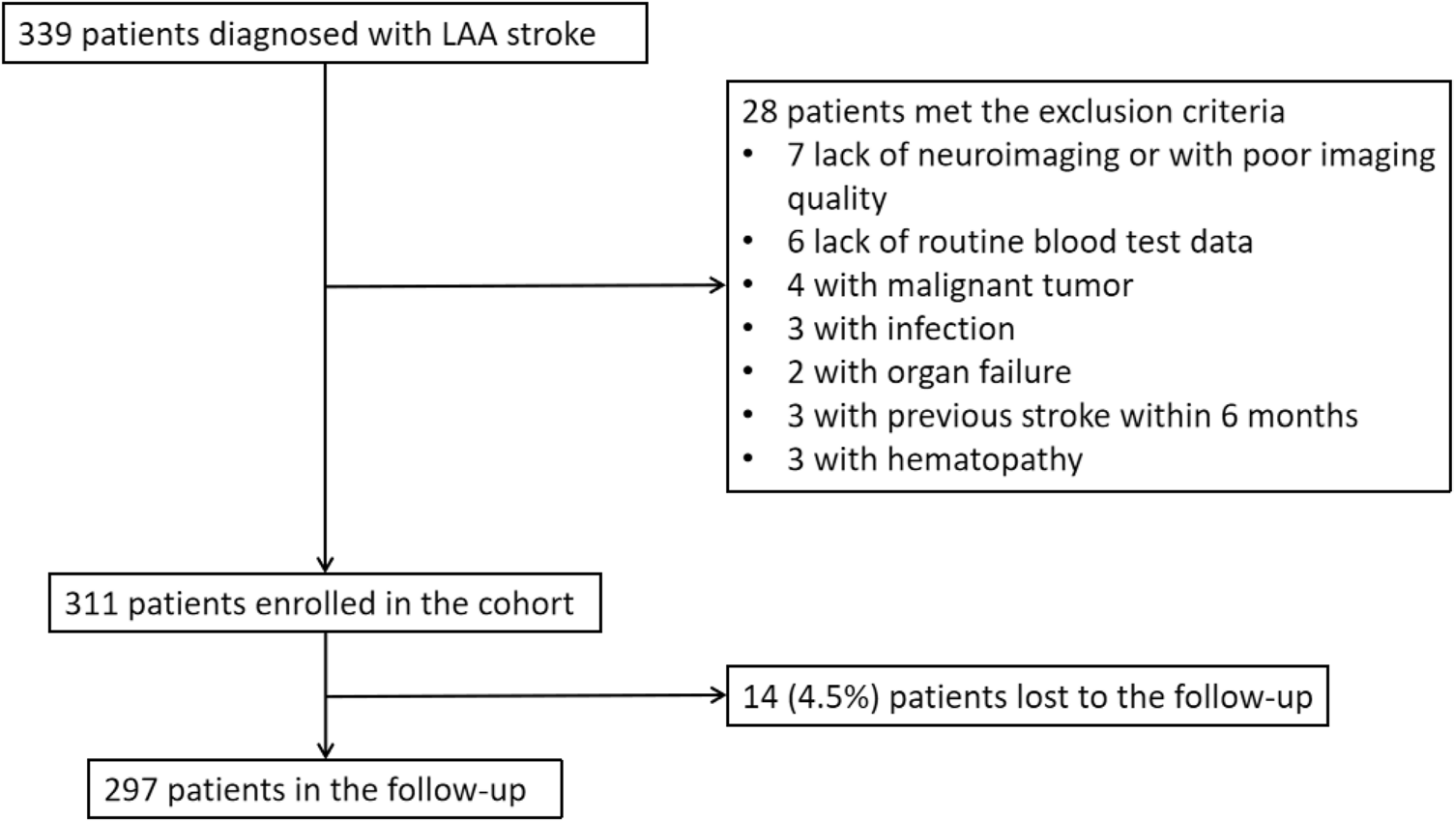
Flow chart of the study selection. *LAA* indicates large artery atherosclerosis.

A total of 339 patients diagnosed with LAA stroke within 7 days of onset were admitted to the stroke unit. Of them, 28 met the exclusion criteria and were excluded from this study, and 311 patients were enrolled in the cohort. Fourteen (4.5%) participants were lost to follow-up, and 297 participants finished the study (Fig. 2). Among these 297 participants kept in the follow-up, 69 (23.2%) had poor 90-day functional outcomes.

### Comparisons of baseline characteristics

All participants were dichotomized into groups with 90-day good (*n* = 228) and poor functional outcomes (*n* = 69). Compared with the good outcome group, the group with poor outcome were older [74.0 (65.0, 79.5) vs. 68.0 (59.0, 74.0) years, *p* < 0.001] and had a higher proportion of previous stroke history (31.9% vs. 20.2%, *p* = 0.043), higher initial NIHSS [6.0 (4.0, 10.5) vs. 2.0 (1.0, 3.0) points, *p* < 0.001], larger infarct size [35.89 (20.49, 58.58) vs. 21.65 (12.88, 42.66) mm, *p* = 0.001], a higher craniocervical AS number [3.0 (2.0, 4.0) vs. 2.0 (1.0, 4.0), *p* < 0.001] and a longer hospital stay [13.0 (9.5, 16.0) vs. 11.0 (9.0, 13.0) days, *p* < 0.001] (Table 1). For the poststroke inflammatory markers, the group with poor outcome had a higher neutrophil count [7.00 (4.75, 8.55) vs. 4.70 (3.70, 5.80) × 10^9^/L, *p* < 0.001], higher neutrophil to lymphocyte ratio (NLR) [4.06 (2.42, 7.29) vs. 2.85 (1.94, 4.26), *p* < 0.001] and lower platelet to neutrophil ratio (PNR) [29.61 (21.88, 41.72) vs. 41.30 (31.36, 55.01), *p* < 0.001] than that with good outcome (Table 2).

**Table 1:** Comparisons of patients with good and poor 90-day functional outcomes.

Clinical characteristics	Total (n = 297)	90-day functional outcome		*p* value
		Good outcome (n = 228)	Poor outcome (n = 69)	
Male, n (%)	177 (59.6)	135 (59.2)	42 (60.9)	0.81
Age (year), median (IQR)	70.0 (60.0, 75.0)	68.0 (59.0, 74.0)	74.0 (65.0, 79.5)	< 0.001*
Time of onset (hour), median (IQR)	24.0 (9.0, 72.0)	24.0 (10.0, 72.0)	24.0 (7.0, 38.0)	0.051
Hypertension, n (%)	237 (79.8)	180 (78.9)	57 (82.6)	0.51
Diabetes mellitus, n (%)	121 (40.7)	90 (39.5)	31 (44.9)	0.74
Smoking, n (%)	122 (41.1)	93 (40.8)	29 (42.0)	0.86
Alcohol consumption, n (%)	90 (30.3)	70 (30.7)	20 (29.0)	0.79
Previous stroke, n (%)	68 (22.9)	46 (20.2)	22 (31.9)	0.043*
Body mass index, median (IQR)	23.42 (23.35, 23.50)	23.42 (23.35, 23.49)	23.44 (23.34, 23.52)	0.55
SBP (mmHg), mean ± SD	156.8 ± 21.7	156.0 ± 21.7	159.4 ± 21.7	0.26
DBP (mmHg), median (IQR)	82.0 (74.5, 91.0)	80.5 (74.3, 92.0)	83.0 (74.5, 80.5)	0.70
Initial NIHSS (point), median (IQR)	2.0 (1.0, 5.0)	2.0 (1.0, 3.0)	6.0 (4.0, 10.5)	< 0.001*
Triglyceride (mmol/L), median (IQR)	1.39 (1.03, 1.95)	1.37 (1.03, 1.94)	1.47 (1.05, 2.01)	0.45
TC (mmol/L), mean ± SD	4.53 ± 1.02	4.49 ± 1.02	4.66 ± 1.04	0.22
HDL-C (mmol/L), median (IQR)	1.03 (0.86, 1.34)	1.02 (0.85, 1.30)	1.12 (0.92, 1.38)	0.15
LDL-C (mmol/L), mean ± SD	2.61 ± 0.83	2.60 ± 0.83	2.67 ± 0.86	0.56
Uric acid (mmol/L), median (IQR)	301.60 (240.75, 391.85)	301.30(241.95, 396.83)	305.20 (234.90, 385.10)	0.55
Glycosylated haemoglobin (mmol/L), median (IQR)	6.30 (5.80, 7.85)	6.20 (5.80, 7.80)	6.40 (5.90, 8.05)	0.87
Homocysteine (mmol/L), median (IQR)	12.26(9.84, 16.01)	12.03 (9.71, 15.41)	12.79(10.04, 17.08)	0.32
Infarct size (mm), median (IQR)	25.16 (14.04, 44.69)	21.65 (12.88, 42.66)	35.89 (20.49, 58.58)	0.001*
Thrombolysis, n (%)	5 (1.7)	3 (1.3)	2 (2.9)	0.33
Craniocervical AS number, median (IQR)	3.0 (1.0, 4.0)	2.0 (1.0, 4.0)	3.0 (2.0, 4.0)	< 0.001*
Hospital stay (day), median (IQR)	9.0 (11.0, 14.0)	11.0 (9.0, 13.0)	13.0 (9.5, 16.0)	< 0.001*
Secondary prevention				
Anti-platelet, n (%)	277 (93.3)	214 (93.9)	63 (91.3)	0.64
Anti-coagulant, n (%)	18 (6.1)	13 (5.7)	5 (7.2)	0.86
Statins, n (%)	294 (99.0)	227 (99.6)	67 (97.1)	0.14

*SBP* systolic blood pressure, *DBP* diastolic blood pressure, *NIHSS* National Institute of Health Stroke Scale, *TC* total cholesterol, *HDL-C* high-density lipoprotein-cholesterol, *LDL-C* low-density lipoprotein cholesterol, *AS* atherosclerotic stenosis.

*p < 0.05 was considered statistically significant.

**Table 2:** Comparisons of poststroke inflammatory markers in patients with different outcomes.

Inflammatory markers	Total (n = 297)	90-day functional outcome		*p* value
		Good outcome (n = 228)	Poor outcome (n = 69)	
Neutrophil count (× 10^9^/L), median (IQR)	5.10 (3.80, 6.60)	4.70 (3.70, 5.80)	7.00 (4.75, 8.55)	< 0.001*
Lymphocyte count (× 10^9^/L), median (IQR)	1.60 (1.20, 2.20)	1.70 (1.20, 2.20)	1.50 (1.10, 2.10)	0.34
Monocyte count (× 10^9^/L), median (IQR)	0.40 (0.30, 0.50)	0.40 (0.30, 0.50)	0.40 (0.30, 0.55)	0.20
Platelet count (× 10^9^/L), mean ± SD	197.58 ± 57.46	198.33 ± 58.67	195.14 ± 53.64	0.69
Hs-CRP (mg/L), median (IQR)	1.50 (0.50, 4.21)	1.50 (0.50, 4.08)	1.60 (0.80, 4.75)	0.43
NLR, median (IQR)	3.05 (2.05, 4.93)	2.85 (1.94, 4.26)	4.06 (2.42, 7.29)	< 0.001*
PNR, median (IQR)	37.90 (28.15, 52.08)	41.30 (31.36, 55.01)	29.61 (21.88, 41.72)	< 0.001*
PLR, median (IQR)	120.00 (79.44, 177.22)	119.62 (77.33, 178.57)	121.90 (83.69, 168.49)	0.78
MLR, median (IQR)	4.17 (3.00, 5.50)	4.40 (3.00, 5.67)	3.67 (2.67, 5.00)	0.084

*Hs-CRP* indicates high-sensitivity C-reactive protein, *NLR* neutrophil to lymphocyte ratio, *PNR* platelet to neutrophil ratio, *PLR* platelet to lymphocyte ratio, *MLR* monocyte to lymphocyte ratio.

*p < 0.05 was considered statistically significant.

### Correlations of the craniocervical AS number and poststroke inflammatory markers with 90-day poor functional outcome

**Figure 3:**
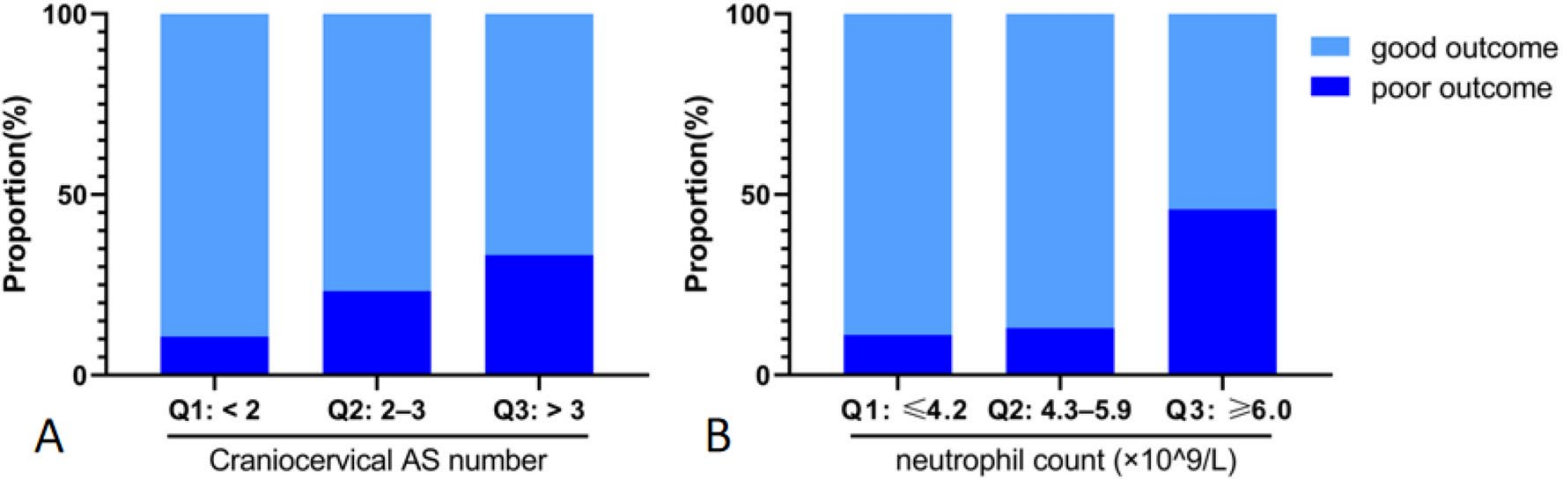
Comparisons of the proportions of 90-day poor functional outcome in the groups, which were divided based on the tertiles of craniocervical AS number and poststroke neutrophil count. (**A**) The proportion of poor outcomes significantly increased with increasing craniocervical AS number (10.7% vs. 23.3% vs. 33.3%, *p* = 0.003). (**B**) The proportion of poor outcomes increased with increasing neutrophil count (11.1% vs. 13.0% vs. 45.9%, *p* < 0.001). The *p* value was calculated by the chi-square test. *p* < 0.05 was considered statistically significant. *AS* indicates atherosclerotic stenosis.

In the multivariable modified Poisson regression and after adjusting for sex, age, time of onset, previous stroke history, initial NIHSS, infarct size and hospital stay, the craniocervical AS number [adjusted relative risk (aRR) = 1.12, 95% confidence interval (CI) 1.04–1.22, *p* = 0.005] was independently associated with 90-day poor functional outcome. Subgroup analyses were performed based on the tertiles of craniocervical AS number (1,3). With an increase in the craniocervical AS number, the proportion of poor outcome significantly increased (10.7% vs. 23.3% vs. 33.3%, *p* = 0.003, Fig. 3A). In the multivariable analysis, compared with the Q_1_ group (AS number < 2), the risk of 90-day poor functional outcome in the Q_2_ group (AS number: 2–3) (aRR = 2.40, 95% CI 1.10–5.26, *p* = 0.029) and Q_3_ group (AS number > 3) (aRR = 3.12, 95% CI 1.39–6.99, *p* = 0.006) increased stepwise (Table 3).

**Table 3:** The relationships of clinical factors with 90-day poor functional outcome in multivariable analyses.

Clinical factors	90-day poor functional outcome			
	Model 1^a^		Model 2^b^	
	Adjusted RR (95% CI)	*p* value	Adjusted RR (95% CI)	*p* value
Craniocervical AS number	1.12 (1.04–1.22)	0.005*	1.06 (0.96–1.16)	0.26
Subgroup analysis				
Q_1_: < 2	Ref		Ref	
Q_2_: 2–3	2.40 (1.10–5.26)	0.029*	2.04 (0.94–4.45)	0.072
Q_3_: > 3	3.12 (1.39–6.99)	0.006*	2.28 (1.03–5.05)	0.05
Neutrophil count	–		1.23 (1.09–1.40)	0.001*
Subgroup analysis				
Q_1_: ≤ 4.2 × 10^9^/L	–		Ref	
Q_2_: (4.3–5.9) × 10^9^/L	–		1.12 (0.49–2.58)	0.78
Q_3_: ≥ 6.0 × 10^9^/L	–		3.16 (1.39–7.14)	0.006*
NLR	–		0.996 (0.94–1.06)	0.89
PNR	–		1.004 (0.99–1.02)	0.60

^a^Adjusted for sex, age, time of onset, previous stroke history, initial NIHSS, infarct size and hospital stay. Inflammatory markers were not included in the model.

^b^Sex, age, time of onset, previous stroke history, initial NIHSS, infarct size, hospital stay and craniocervical AS number, neutrophil count, NLR and PNR were simultaneously included in the model.

*AS* indicates atherosclerotic stenosis, *Ref* reference, *NLR* neutrophil to lymphocyte ratio, *PNR* platelet to neutrophil ratio.

*p < 0.05 was considered statistically significant.

Then, we added the neutrophil count, NLR and PNR into the modified Poisson regression model. After adjusting for the aforementioned confounders, neutrophil count (aRR = 1.23, 95% CI = 1.09–1.40, *p* = 0.001) took the place of craniocervical AS number as an independent predictor for 90-day poor functional outcome. The NLR and PNR were not independently associated with poor outcome. All participants were divided into 3 groups based on tertiles of the neutrophil count [(4.2, 5.9) × 10^9^/L]. The proportions of 90-day poor functional outcome among these three groups were significantly different (11.1% vs. 13.0% vs. 45.9%, *p* < 0.001, Fig. 3B). In the multivariable analysis, compared with the Q_1_ group (neutrophil count ≤ 4.2 × 10^9^/L), the risk of 90-day poor functional outcome in the Q_3_ group (neutrophil count ≥ 6.0 × 10^9^/L) was increased (aRR = 3.16, 95% CI = 1.39–7.14, *p* = 0.006) (Table 3).

**Figure 4:**
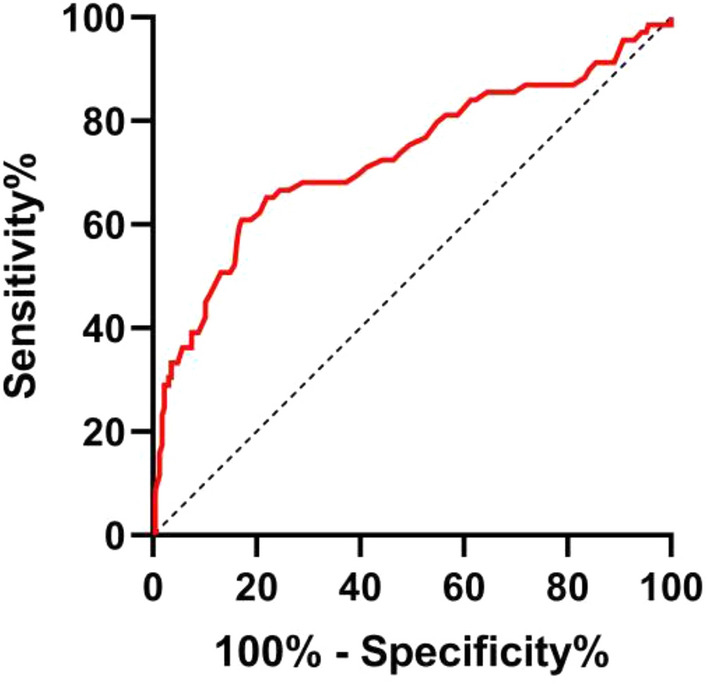
The ROC curve of poststroke neutrophil count in predicting the poor outcome of patients with LAA stroke. *LAA* indicates large-artery atherosclerosis.

In predicting the poor outcome of all participants, the AUC of the poststroke neutrophil count was 0.73 (95% CI = 0.65–0.81, *p* < 0.001), and the cutoff was 6.35 × 10^9^/L, with a sensitivity of 60.9% and a specificity of 82.9% (Fig. 4).

### Correlations of poststroke inflammatory markers and craniocervical AS number

Patients were divided into 3 groups according to the tertiles of the craniocervical AS number. Along with the increase in the craniocervical AS, the neutrophil count and the NLR increased, whereas the PNR decreased in a dose-dependent manner. In addition, the monocyte count also showed a significant difference among these 3 groups (Table 4).

**Table 4:** Comparisons of inflammatory markers among patients with different AS numbers.

Inflammatory markers	Craniocervical AS number			
	Q_1_ group (< 2)	Q_2_ group (2–3)	Q_3_ group (> 3)	*p* value
Neutrophil count (× 10^9^/L), median (IQR)	4.30 (3.30, 5.50)	5.10 (4.05, 6.35)	5.70 (3.70, 7.85)	0.001*
Lymphocyte count (× 10^9^/L), median (IQR)	1.50 (1.20, 2.20)	1.70 (1.20, 2.20)	1.60 (1.05, 2.25)	0.59
Monocyte count (× 10^9^/L), median (IQR)	0.40 (0.30, 0.50)	0.40 (0.30, 0.60)	0.40 (0.30, 0.50)	0.011*
Platelet count (× 10^9^/L), mean ± SD	191.53 ± 56.46	202.89 ± 60.03	195.10 ± 54.52	0.24
Hs-CRP (mg/L), median (IQR)	1.60 (0.50, 3.20)	1.50 (0.50, 3.89)	1.60 (0.70, 6.55)	0.28
NLR, median (IQR)	2.93 (1.67, 4.00)	3.05 (2.05, 4.71)	3.24 (2.25, 6.60)	0.027*
PNR, median (IQR)	41.84 (34.52, 52.58)	38.10 (29.53, 50.78)	31.38 (23.17, 54.66)	0.009*
PLR, median (IQR)	118.82 (76.58, 171.54)	121.74 (84.80, 175.57)	118.33 (77.79, 203.00)	0.73
MLR, median (IQR)	4.67 (3.00, 6.75)	4.00 (2.75, 5.25)	4.00 (3.00, 5.58)	0.071

*AS* indicates atherosclerotic stenosis, *Ref* reference, *Hs-CRP* high-sensitivity C-reactive protein, *NLR* neutrophil to lymphocyte ratio, *PNR* platelet to neutrophil ratio, *PLR* platelet to lymphocyte ratio, *MLR* monocyte to lymphocyte ratio.

*p < 0.05 was considered statistically significant.

In the univariable analysis, age, history of diabetes mellitus and previous stroke, level of glycosylated hemoglobin, infarct size, and previous use of antidiabetic agents were significantly different among the 3 groups (Supplementary Table S1). In the multivariable ordinal logistic regression analysis, after adjusting for the abovementioned factors and sex, body mass index and previous use of statins, the neutrophil count was significantly correlated with the craniocervical AS number [adjusted odds ratio (aOR) = 1.41, 95% CI 1.16–1.72, *p* = 0.001] (Table 5).

**Table 5:** The relationships of poststroke inflammatory markers with the craniocervical AS number in multivariable ordinal logistic analyses.

Inflammatory markers	Craniocervical AS number			
	Crude OR (95% CI)	*p* value	Adjusted OR^a^ (95% CI)	*p* value
Neutrophil count	1.30 (1.16–1.45)	< 0.001*	1.41 (1.16–1.72)	0.001*
PNR	1.18 (0.99–1.26)	0.062	1.06 (0.93–1.20)	0.41
NLR	1.10 (1.02–1.19)	0.011*	1.02 (0.92–1.12)	0.76
Monocyte count	1.20 (0.75–1.92)	0.45	0.85 (0.49–1.48)	0.56

^a^Age, sex, diabetes mellitus and previous stroke history, body mass index, glycosylated hemoglobin, infarct size, previous use of antidiabetic agents and statins, neutrophil and monocyte counts, PNR and NLR were simultaneously included in the model.

*AS* indicates atherosclerotic stenosis, *PNR* platelet to neutrophil ratio, *NLR* neutrophil to lymphocyte ratio.

*p < 0.05 was considered statistically significant.

## Discussion

The present study found that the craniocervical AS number was independently correlated with 90-day poor functional outcome of patients with LAA stroke after adjusting for clinical factors except the poststroke inflammatory markers. After the inclusion of poststroke inflammatory markers into the multivariable Poisson regression model, neutrophil count took the place of craniocervical AS number as an independent predictor of 90-day poor functional outcome (Table 3). Meanwhile, the poststroke neutrophil count was positively associated with the craniocervical AS number in patients with LAA stroke (Tables 4 and 5).

In our previous work, we found that craniocervical atherosclerotic severity is an independent predictor of poor functional outcomes in patients with LAA stroke^8^. However, in that work, we did not adjust for poststroke inflammatory markers in the multivariable analyses that explored the association between craniocervical atherosclerotic severity and the functional outcome of the study subjects. In the present study, the poststroke inflammatory markers and craniocervical AS number were simultaneously included in the multivariable analyses, and we demonstrated that poststroke neutrophil count, instead of the craniocervical AS number, is an independent predictor for 90-day poor functional outcome of patients with LAA stroke. This suggests that the poststroke neutrophil count is a stronger predictor of poor outcome in patients with LAA stroke than craniocervical atherosclerotic severity.

We subsequently explored the correlation of poststroke inflammatory markers with the craniocervical AS number in the study subjects. We found that only poststroke neutrophil count was positively associated with craniocervical AS number after adjusting for all confounders. This result suggests that neutrophils in peripheral circulation are upregulated with increasing craniocervical AS number after the onset of LAA stroke. Given that neutrophils enlarge the infarct size and worsen stroke severity through the release of toxic cytokines^11–13^, the positive relationship between poststroke neutrophil count and craniocervical AS number demonstrated in this study is probably a key factor mediating the adverse effect of craniocervical AS on the functional outcome of patients with LAA stroke. For LAA stroke patients with a severe craniocervical atherosclerotic burden, a large amount of neutrophils may migrate into the infarcts after the onset of stroke and introduce secondary injury to the neurons, ultimately worsening the functional outcome of these patients.

Atherosclerosis is a chronic inflammatory disease offending the vascular wall. In the process of atherosclerotic formation, neutrophils adhere to the endothelium and promote the aggregation of monocytes, thereby aggravating inflammation of the vascular wall. Furthermore, neutrophils can destabilize atherosclerotic plaques by releasing proteinase^31^. On the basis of this long-term chronic inflammation caused by neutrophils, patients with a severe craniocervical atherosclerotic burden may experience strong neutrophil mobilization after the onset of stroke^19^. This is probably the reason for the positive relationship of poststroke neutrophil count with craniocervical AS number in patients with LAA stroke. Meanwhile, we have also demonstrated that poststroke neutrophil count could to some extent represent the craniocervical atherosclerotic severity of patients with LAA stroke, which is in line with the conjecture of Buck BH and colleagues^27^.

A previous study reported that poststroke neutrophil count is significantly associated with infarct size in patients with ischemic stroke due to the inflammatory response caused by the infarct lesion^27^. In the present study, we measured the infarct sizes of all participants and considered this parameter as a confounder that might influence the relationship between the neutrophil count and craniocervical AS number. In multiple logistic regression models, after adjusting for infarct size, we found that neutrophil count was still independently associated with craniocervical AS number (Table 5). This suggests that in addition to the dependence upon the infarct size, the poststroke neutrophil count is also associated with craniocervical AS number.

This study provides some clinical guidance. For patients with LAA stroke, the evaluation of poststroke neutrophil count is likely to be a stronger predictor of poststroke disability than the evaluation of craniocervical AS. This contributes to early identification in clinical practice of LAA stroke patients with a high risk of poststroke disability. In the ROC analysis of the present study, the cutoff value of poststroke neutrophil count in predicting poor outcome was 6.35 × 10^9^/L. For LAA stroke patients with a poststroke neutrophil count > 6.35 × 10^9^/L, anti-inflammatory therapy, especially in patients with elevated neutrophils, is probably beneficial for the clinical outcomes of these patients. Further clinical trials are needed to validate this conjecture.

The present study has some limitations. First, this study is a single-center study, and the majority of the participants are from the Chinese Han population. Caution must be taken when the results of this study are generalized to other regions or populations. Second, infarct size is represented by the largest diameter of ischemic lesion in the present study, instead of being calculated with specific software. However, a previous study corroborated that the largest ischemic lesional diameter could relatively reflect the infarct volume^32^. Finally, this study merely analyzed leukocyte counts acquired from routine blood tests without analyzing inflammatory cytokines that can also reflect the poststroke inflammatory response, such as interleukin and tumor necrosis factor. A future study could be conducted to investigate the relationships between these cytokines and craniocervical atherosclerotic burden after the onset of LAA stroke.

## Conclusion

This study has demonstrated that the poststroke neutrophil count is a valuable predictor of 90-day poor functional outcome in patients with LAA stroke. The poststroke neutrophil count is significantly correlated with craniocervical AS number in these patients. This study preliminarily elucidates the mechanism underlying the adverse effect of craniocervical AS on the functional outcome of patients with LAA stroke and provides guidance for the clinical treatment of these patients.

## Supplementary Information

Supplementary Information 1.

Supplementary Information 2.

Supplementary Table S1.

## Data Availability

All data generated or analyzed during this study are included in this published article (and its Supplementary Information files).
