# Supplementary material for: Poststroke neutrophil count is predictive of the outcomes of large-artery atherosclerotic stroke and associated with craniocervical atherosclerosis
Source: Sci Rep. 2023 Jul 17;13:11486. doi: 10.1038/s41598-023-37815-5 (PMC10352250; doi:10.1038/s41598-023-37815-5)
Supplement: Supplementary file 3 — Supplementary Table S1. [file 41598_2023_37815_MOESM3_ESM.pdf]

Supplementary Table S1 Comparisons of patients divided based on the tertiles of post-stroke neutrophil count

Clinical characteristics	Neutrophil count (×10^9/L)			*p* value
	Q_1: ≤ 4.2 (n=99)	Q_2: 4.3–5.9 (n=100)	Q_3: ≥ 6.0 (n=98)	
Male, n (%)	57 (57.6)	66 (66.0)	54 (55.1)	0.26
Age (year), median (IQR)	68.0 (59.0, 75.0)	70.0 (60.0, 75.0)	70.0 (62.5, 76.0)	0.64
Time of onset (hour), median (IQR)	24.0 (8.0, 72.0)	24.0 (10.0, 72.0)	24.0 (10.0, 49.5)	0.80
Hypertension, n (%)	77 (77.8)	80 (80.0)	80 (81.6)	0.80
Diabetes mellitus, n (%)	34 (34.3)	48 (48.0)	39 (39.8)	0.14
Smoking, n (%)	40 (40.4)	46 (46.0)	36 (36.7)	0.41
Alcohol consumption, n (%)	28 (28.3)	30 (30.0)	32 (32.7)	0.80
Previous stroke, n (%)	14 (14.1)	23 (23.0)	31 (31.6)	0.014*
Body mass index,median (IQR)	23.41 (23.33, 23.50)	23.44 (23.37, 23.49)	23.42 (23.35, 23.49)	0.48
SBP (mmHg), mean ± SD	155.3±22.0	157.1±21.6	158.1±21.7	0.67
DBP (mmHg), median (IQR)	80.0 (75.0, 90.0)	81.5 (74.0, 92.0)	82.0 (75.0, 90.3)	0.80
Initial NIHSS (point), median (IQR)	2.0 (1.0, 5.0)	2.0 (1.0, 4.0)	2.0 (1.0, 6.3)	0.034*
Triglyceride (mmol/L), median (IQR)	1.29 (0.95, 1.80)	1.39 (1.12, 1.99)	1.49 (1.07, 2.04)	0.037*
TC (mmol/L), mean ± SD	4.27±0.94	4.74±1.02	4.58±1.06	0.004*
HDL-C (mmol/L), median (IQR)	1.05 (0.85, 1.39)	1.02 (0.86, 1.24)	1.06 (0.86, 1.33)	0.76
LDL-C (mmol/L), mean ± SD	2.37±0.73	2.77±0.84	2.70±0.87	0.001*
Uric acid (mmol/L), median (IQR)	289.80 (236.50, 362.90)	299.50 (238.13, 389.75)	336.00 (259.90, 403.33)	0.18
Glycosylated hemoglobin (mmol/L), median (IQR)	6.00 (5.70, 7.20)	6.25 (5.90, 8.18)	6.70 (5.90, 8.20)	0.021*
Homocysteine (mmol/L), median (IQR)	12.05 (9.53, 15.00)	11.59 (9.72, 16.03)	12.87 (9.87, 16.94)	0.55
Infarct size (mm), median (IQR)	21.34 (12.80, 43.40)	22.99 (13.53, 43.63)	32.03 (16.42, 48.65)	0.14
Craniocervical AS number, median (IQR)	2.0 (1.0, 4.0)	2.0 (2.0, 3.0)	3.0 (2.0, 4.0)	0.022*
Previous medication				
Antihypertensive agents, n (%)	68 (68.7)	76 (76.0)	72 (73.5)	0.50
Antidiabetic agents, n (%)	28 (28.3)	46 (46.0)	34 (34.7)	0.031*
Anti-platelet, n (%)	11 (11.1)	7 (7.0)	14 (14.3)	0.25
Statins, n (%)	11 (11.1)	6 (6.0)	13 (13.3)	0.22

Abbreviations: SBP indicates systolic blood pressure; DBP, diastolic blood pressure; NIHSS, National Institute of Health Stroke Scale; TC, total cholesterol; HDL-C, high-density lipoprotein-cholesterol; LDL-C, low-density lipoprotein cholesterol; AS, atherosclerotic stenosis.

* $p < 0.05$ was considered statistically significant.
